# Detect Acute Porphyrias in Emergency Departments (DePorED) – a pilot study

**DOI:** 10.1186/s13023-023-02768-5

**Published:** 2023-06-12

**Authors:** Eva Diehl-Wiesenecker, Sabine Blaschke, Nils Wohmann, Ilja Kubisch, Thomas Stauch, Mona Mainert, Franziska Helm, Sabine von Wegerer, David Pittrow, Jorge Frank, Ulrich Stölzel, Rajan Somasundaram

**Affiliations:** 1 Department of Emergency Medicine and Porphyria Clinic, Charité University Medicine Berlin, Freie Universität Berlin and Humboldt-Universität zu Berlin, Hindenburgdamm 30, 12203 Berlin, Germany; 2 Emergency Department, University Medical Center Göttingen, Göttingen, Germany; 3 Department of Dermatology, Venerology and Allergology, University Medical Center, Göttingen, Germany; 4 Department of Internal Medicine II and Saxony Porphyria Center, Klinikum Chemnitz, Chemnitz, Germany; 5 Labor Volkmann, Karlsruhe, Germany; 6 Institute for Clinical Pharmacology, Technical University of Dresden (TUD), Dresden, Germany; 7 Innovation Center Real World Evidence, GWT-TUD, Dresden, Germany; 8 Berliner Leberring e.V, Berlin, Germany

**Keywords:** Acute Porphyrias, Screening, Emergency Department, Awareness, Education, Abdominal Pain

## Abstract

**Background:**

Acute porphyrias (APs) are a group of rare metabolic diseases related to a disturbed heme biosynthesis. Symptoms may first occur as life threatening attacks, comprising abdominal pain and/or variable neuro-psychiatric symptoms, thus leading to presentation in emergency departments (ED) first. Due to the low prevalence, diagnosis of AP is often missed, even after readmission to the ED. Therefore, strategies are needed to consider APs in ED patients with unexplained abdominal pain, especially since early and adequate treatment will avoid an unfavorable clinical course. Aim of this prospective study was to investigate the prevalence of APs in ED patients and thus, addressing feasibility of screening for rare diseases, such as APs in the real life setting.

**Methods:**

From September 2019 to March 2021, patients presenting to the ED of three German tertiary care hospitals with moderate to severe prolonged abdominal pain (Visual Analog Scale, VAS > 4 out of 10 points) not otherwise explained were screened and prospectively enrolled. In addition to standard of care (SOC) diagnostics a blood and urine sample for plasma fluorescence scan and biochemical porphyrin analysis were sent to a certified German porphyria laboratory.

**Results:**

Overall, of 653 screened patients, 68 patients (36 females; mean age 36 years) were included for biochemical porphyrin analysis. No patient with AP was detected. The most frequent discharge diagnoses included “abdominal and digestive symptoms” (n = 22, 32%), “gastrooesophageal diseases” (n = 18, 27%), “infectious bowel disease” (n = 6, 9%) and “biliopancreatic diseases” (n = 6, 9%). Although not primarily addressed, we observed an increase in knowledge of the ED staffs at all study sites regarding our screening algorithm and thus, awareness for APs.

**Conclusions:**

To the best of our knowledge, we performed the first prospective screening project for APs in the ED. Although we detected no patient with AP in this study, we demonstrated the feasibility of a multicenter screening process for APs by building up a well-working infrastructure comprising laboratory testing as well as data management. This enables the set-up of a larger scale revised follow-up study with a central focus on structured education, thus, possibly acting as blueprint for other rare diseases.

**Supplementary Information:**

The online version contains supplementary material available at 10.1186/s13023-023-02768-5.

## Introduction

Acute porphyrias (APs) are a group of rare metabolic diseases. For each AP a well characterized enzyme of the heme biosynthesis pathway is deficient. Simultaneously the activity of the first enzyme of the sequence – 5 aminolevulic-acid-synthase 1 (ALAS 1) – will be upregulated as a compensatory mechanism, which results in accumulation of porphyrins and their precusors. In total, 8 types of porphyrias are distinguished: 4 acute and 4 non-acute porphyrias, the latter often presenting with sun light sensitivity, whereas patients with APs may display a plethora of different and acute symptoms (see below). There are three autosomal dominant inherited forms of APs: acute intermittent porphyria (AIP), variegate porphyria (VP) and hereditary coproporphyria (HCP), further the autosomal recessive inherited and very rare 5-aminolevulinic-acid (ALA)-dehydratase-deficient porphyria (ADP). To date, the genetic basis of all porphyrias has been unraveled and distinct pathogenic variants have been described. But penetrance is very low (estimated as approximately 1% in general population). Thus, about > 99% of gene carriers remain asymptomatic throughout life and the prevalence of symptomatic AP patients in the general population, e.g. in Germany between 1:100.000 to 1:200.000, may underestimate the number of gene carriers [1–3].

Patients with APs may experience acute attacks with life-threatening neuro-psychiatric, abdominal and cardiovascular symptoms. VP and HCP can also present with cutaneous manifestations such as blistering lesions on sunlight-exposed skin areas. Acute attacks, usually manifest after puberty and can be induced by all forms of stress and/or cytochrome P450 inducing factors, e.g. surgery, infection, fasting, smoking, excessive alcohol consumption, sexual hormones, xenobiotics, including several drugs [1, 2, 4]. More than 75% percent of patients with acute attacks need to be hospitalized [5]. Therefore, in patients with an acute attack, who usually present to an ED first, immediate diagnosis and treatment is required.

Diagnosis in symptomatic AP patients can be made effectively and cost-efficiently via a simple spot urine analysis [6]. If available and especially in patients with severe symptoms presenting to the ED, plasma fluorescence analysis is reliable for an even faster screening, but positive results should be confirmed by urine spot analysis [1]. Treatment of acute attacks includes identification and avoidance of triggering factors, e.g. by discontinuing porphyrinogenic medication, carbohydrate loading with oral or intravenous glucose in milder attacks (without paresis and hyponatremia) and application of intravenous hemin when neurological symptoms occur. Furthermore, a supportive therapy with e.g. analgesics or antiemetics is necessary [1–3, 7].

Early diagnosis and adequate treatment, e.g. with hemin, in the ED can prevent severe courses, irreversible damage and fatal consequences such as mechanical ventilation, seizures or tetraplegia [4, 5, 7]. However, there is an unmet need for emergency physicians (EPs) to appropriately diagnose rare diseases in time. EPs are trained to pragmatically allocate patients into common categories allowing the rapid identification of patients with common and time-critical conditions.

Particularly the hallmark symptom of recurrent abdominal pain may create some difficulties for EPs. Since abdominal pain is caused by a multitude of more common chronic conditions, uncommon diseases are often not considered and missed. Despite comprehensive workups in the ED and after in-hospital admission, diagnoses often remain inconclusive. Unnecessary, exploratory surgical interventions are not uncommon [8–10]. Thus, even in severe symptomatic AP patients (“acute attack”) diagnosis is often missed resulting in a diagnostic delay of up to 15 years[1, 5].

Since a large number of patients present to German EDs per year (around 20 million patients/year; approx. 10% with abdominal pain), EDs may be appropriate for screening strategies of rare diseases, such as APs [11]. Thus, in this multicenter prospective screening pilot study for APs, we aimed to detect patients with so far unknown APs and to investigate the feasibility of such a screening approach in the real life setting of an ED.

## Methods

### Study design and participant selection

A prospective, observational study was conducted at the German EDs Charité University Medicine Berlin (Campus Benjamin Franklin), University Medical Center Göttingen and Klinikum Chemnitz (all level 3) from September 2019 to March 2021. Due to the ongoing pandemic with a decline of patient numbers in EDs except for COVID-19, reduction of resources in the EDs and the advice from hospital administration to focus study activity mainly on COVID-19-studies, the study was terminated preliminary.

All patients presenting to the ED with abdominal pain were screened after being triaged. Patients with the following inclusion criteria were prospectively enrolled: (1) severe and prolonged abdominal pain (duration ≥ 4 h, intensity on visual analog scale ≥ 7 at presentation, in order to not miss no single patient with AP, patients with pain ≥ 4 have were also included since January 2021), (2) age ≥ 18 and ≤ 75 and (3) written informed consent. Exclusion criteria were abdominal pain of known or other probable cause after ED-workup, missing written informed consent, previous diagnosis of AP and < 18 years of age. No other exclusion criteria (including COVID-19) were defined, since we aimed to include a broad population in this screening and awareness study. The study was approved by the institutional ethics review boards of Berlin (EA4/099/19), Chemnitz (EA4/099/19) and Göttingen (14/1/20 Ü).

### Team training

In all participating centers team training sessions (approx. 20–30 min) were performed for the physicians and nurses in the ED, addressing the aims of the study, the study design, the inclusion and exclusion criteria as well as a basic transfer of knowledge of porphyria pathophysiology and symptoms. Especially by pointing to patients’ potentially worse outcome, if AP patients were not diagnosed in time, the need of early diagnosis in the ED was emphasized. Furthermore, during the ongoing study awareness of the staff was supported by study nurses and by displaying posters in the ED, summarizing the essentials of the study.

### Probe sampling

In addition to standard of care (SOC) diagnostics for patients with abdominal pain, such as blood samples for complete blood count with differential, C-reactive protein, lipase, lactate, aminotransferases, alkaline phosphatase, and bilirubin, glucose, sodium, potassium, calcium, creatinine, urine analysis, abdominal ultrasound, electrocardiogram, and CT-scan if needed, pregnancy test in females of childbearing age [12] a heparinized blood and a spot urine sample were collected from patients enrolled in the study, wrapped to keep them dark and sent to the central certified porphyria laboratory (MVZ Labor Volkmann, Karlsruhe). Samples collected at night shifts or weekends were processed as described above, stored at room temperature and sent on the next weekday to the laboratory. At first, plasma fluorescence scans of the plasma sample were performed. In case of a positive result or impossibility of plasma fluorescence scanning (e.g. due to hemolysis or hyperbilirubinemia), 5-aminolevulinic acid (5-ALA) and porphobilinogen (PGB) concentrations in urine were measured. Since in symptomatic patients with AP the latter analyses are effective and time-efficient to diagnose AP, no mutation analysis for AP was initiated in the EDs [1–3].

### Data management

Each patient was assigned a number, which only the attending physician could link with the person on the basis of a patient identification list (pseudonymisation), which remained at the respective hospital. The data protection provisions, in particular the EU General Data Protection Regulation (GDPR), were strictly observed. Patients’ initials or exact date of birth were not entered in the database. Birth year and sex were only documented on the laboratory vials.

Laboratory data (including patient ID, birth year, sex) were sent as CSV files from the central laboratory to the Gesellschaft für Wissenschafts- und Technologietransfer at Technical University of Dresden (GWT-TUD). The data were automatically checked for completeness and plausibility and stored on the GWT server. These pseudonymised laboratory data were accessible for GWT-TUD and persons commissioned by it (project management, data manager, monitor, and statistician), the sponsor of the study (Alnylam Pharmaceutics Ltd.), and representatives of the supervisory authorities or ethics committees. Patients were requested to agree to this in the declaration of consent.

### Statistical analysis

Descriptive statistical analysis was performed with all the pseudomized data at the Berlin Center. SPSS statistics (Version 28, IBM corporation, New York, USA) was used.

## Results

### Characteristics of study participants

**Fig. 1:**
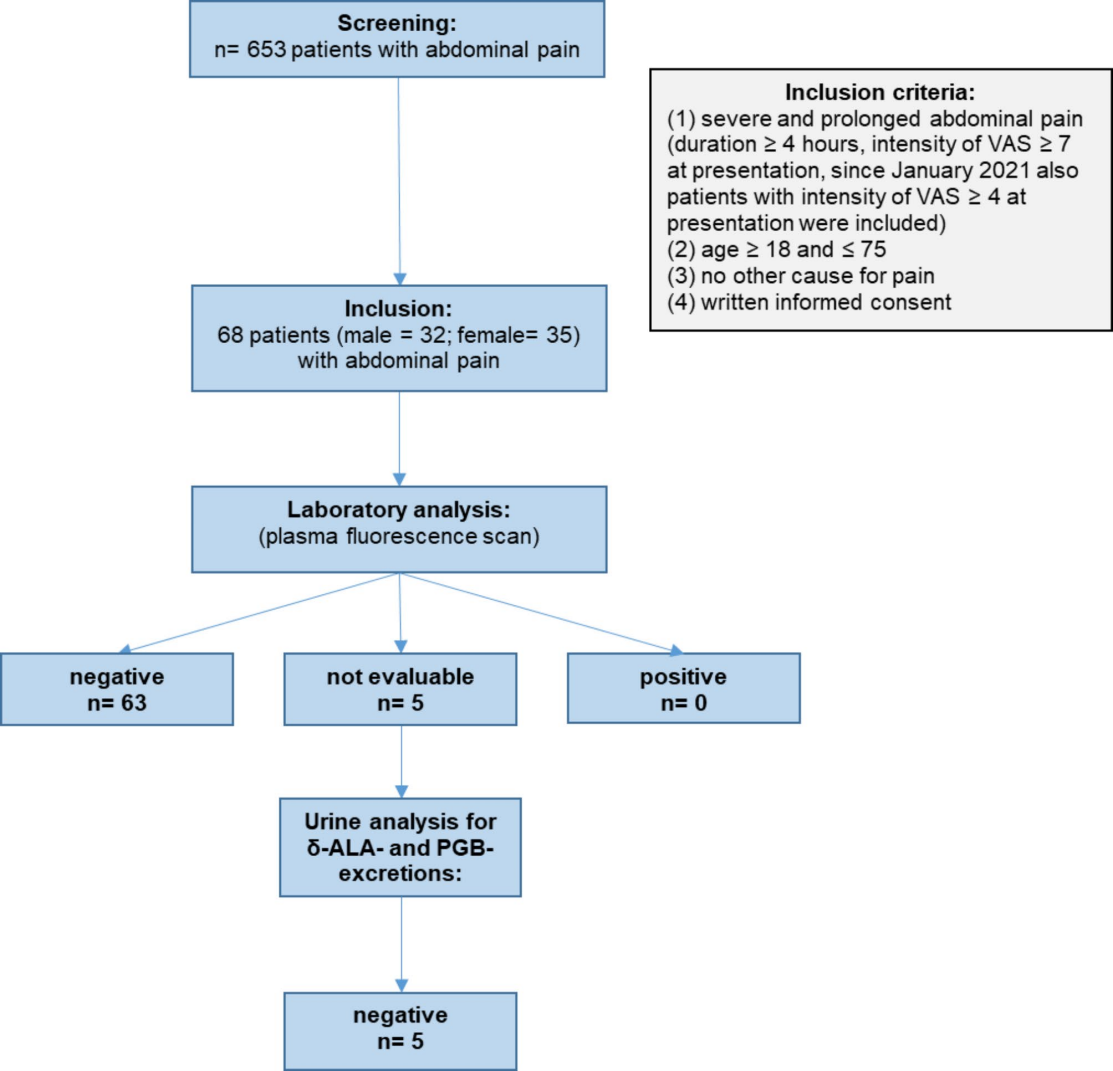
Flow Chart of enrollment and laboratory analytic process

In total 653 patients were screened for inclusion in the study. The enrolled cohort comprised 68 patients, 36 (53%) female and 32 (47%) male subjects, with a mean age of 39 years. Five samples could not be evaluated due to sampling errors (Fig. 1).

All of the patients presented with abdominal pain (inclusion criterion). Two patients (3%) revealed additional hyponatremia, which might be associated with APs, eight patients (12%) presented with additional neurologic symptoms to the ED (Table 1).

### Laboratory analysis

Among the analyzed serum samples, no emission signals could be detected in the range between 580 and 650 nm on excitation with 405 nm (plasma fluorescence scan). In 5 blood samples plasma fluorescence scan was not evaluable due to sample properties/-specifications, therefore, urine analysis for 5-ALA- and PGB-excretions was performed alternatively. These determinations gave invariably inconspicuous results below the corresponding diagnostic cut-off values, thus, no case of AP could be recorded (Fig. 1).

### Feasibility

Sample collection and sending was safely and easily feasible due to tube identification with self-adhesive barcode labels (ID on the request form as well). Urine spot samples and blood samples were taken simultaneously and protected from light immediately after retrieval. The samples were sent together with a request form specially designed for this study.

At the laboratory site an IT-based procedure was established leading to automated insertion of requests for porphyrin precursor determination in the corresponding urine sample in case of missing or failed evaluation of plasma fluorescence scanning. All samples were archived after processing at -20 °C.

### Social-educative effects

Although not primarily addressed and thus, not systematically evaluated, a gain of awareness in the ED staff (physicians and nurses) concerning our screening algorithm was observed at all participating study sites. Interestingly, even after the end of our study we registered, that APs were taken more often into account as a differential diagnosis in abdominal pain of unclear origin.

### Discharge diagnoses

Among the 68 patients, the most frequent discharge diagnoses were unspecific and symptom-related: “abdominal and digestive symptoms” (n = 22, 32%), “oesophageal, gastric and duodenal diseases” (n = 18, 27%), “infectious bowel disease” (6, 9%) and “biliary and pancreatic diseases” (n = 6, 9%). Four patients (6%) were discharged without final diagnosis.

**Fig. 2:**
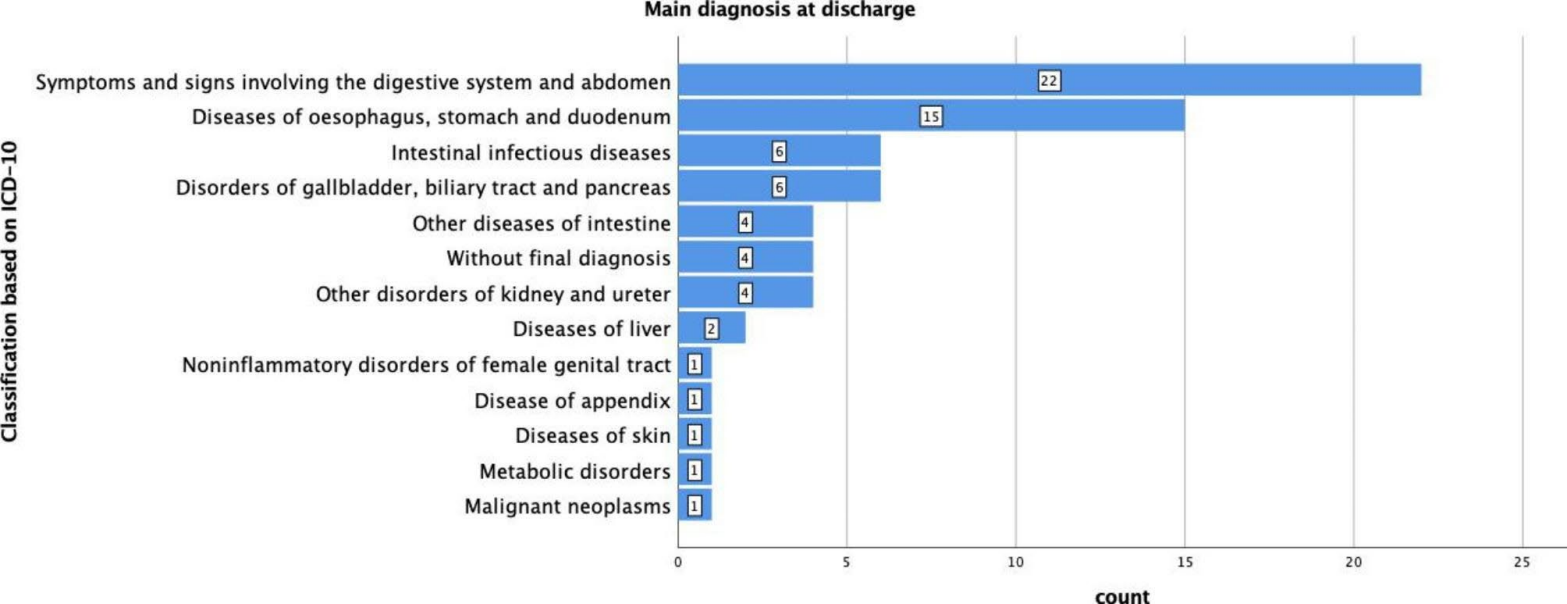
Differential diagnoses of acute abdominal pain in three tertiary care EDs as assessed by ICD-10 diagnostic group

All discharge diagnoses are depicted in Fig. 2. The classification is based on the German version of International Statistical Classification of Diseases and Related Health Problems, version 10 (ICD-10).

**Table 1:** Patient details of included individuals

Characteristics	n (%)
**Total patients** Males Females Unknown	68 32 (47.1) 35 (51.5) 1 (1.5)
**Age** Mean Median Range	**(in years)** 38.6 36.5 18–75
**Associated Symptoms**	
Neurological Hyponatremia	8 (11.8) 2 (2.9)

## Discussion

To the best of our knowledge, we present the results of the first multicenter prospective screening project for APs in a real life ED setting. Although no patient with an AP was detected, we demonstrated the feasibility of such a study addressing a rare disease.

In a total number of 68 patients included, we did not find any case of AP. Since this pilot study had to be terminated prematurely due to the COVID-19 pandemic a relatively small number of 653 patients could be screened and finally 68 patients fulfilled the inclusion criteria for analyzing porphyrins. Thus, this result was expected.

Since an acute attack of APs is defined by porphyria symptoms, such as abdominal pain that is not explained otherwise, and at least a five-fold upper normal elevated concentration of urinary 5-ALA- and PGB [1–3] both of them measured in case of a suspicious fluorescence scan, the likelihood that we missed any patient with AP in our cohort is low.

Psysicochemical or biochemical diagnostics, respectively, as outlined above are known to be sufficient to not miss a symptomatic patient (100% sensitivity in the absence of coarse preanalytical faults) with an AP, so we abstained from even more time-consuming genetic testing. Moreover, restrictions from our ethics committee did not allow for mutational analyses in this study. However, in further studies genetic testing could be included to show the frequency of AP variants in patients with unexplained abdominal complaints.

The mean age of our cohort was consistent with the described mean onset of symptoms in patients with AP. Certainly, the large proportion of male participants wasn´t in line with data reporting women as being more often affected with overt AP [5, 13].

Yet, this pilot study showed the feasibility of such a screening project. A well-functioning infrastructure was set up comprising high quality laboratory analysis and data management, now allowing recruitment of even more study sites. Rapid spot urine sampling and dispatch of darkened vials for plasma and urine analysis by one of two certified German porphyria laboratories were shown to be feasible even in a busy ED setting.

Although testing for APs in patients with abdominal pain is not a first line recommendation in the ED, it should be performed even more regularly, especially in patients with recurrent abdominal pain of unclear origin [8, 14, 15]. As outlined in the introduction, early detection of AP result in less morbidity and mortality for affected patients. Furthermore, adequate therapeutic regimens, e.g. hemin, could be applied in acute phases of porphyrias if the disease is detected as early as possible in these patients [1–3].

At all study sites it was observed, that the education and promotion of knowledge of the entire ED staff regarding a screening algorithm for the potential diagnosis of AP led to an increased awareness and interest for APs in general. However, this was not the primary objective of our study and thus, not statistically measured. Nevertheless, an important take home point is, that structured and brief summaries of symptoms, complications and possible screening raise awareness regarding rare diseases even in a busy ED.

Another secondary effect was the establishment of a well-working national porphyria network among the participating centres, including the EDs. Such networks are an ultimate precondition for detection and treatment of rare diseases. Starting with the “jour fixe” concerning this study, video and live meetings now take place on a regular basis covering case discussions, education and research issues. Furthermore, our approach and results concerning feasibility demonstrate, that inclusion of emergency departments with their high patient volumes in such networks might be helpful to detect certain rare diseases even more early.

Moreover, our results list up discharge diagnoses other than AP to better understand common and rare causes of abdominal pain. This is in line to another large ED study, where the most frequent main hospital diagnoses of patients presenting with abdominal pain were of gastrointestinal origin, although the most frequent discharge diagnosis was acute pancreatitis (9.4%) [11]. A distinct knowledge of differential diagnoses for the nonspecific hallmark symptom abdominal pain should be of utmost interest for EPs, as timely recognition and accurate diagnosis is crucial to reduce both unnecessary diagnostic interventions and unnecessary pain and suffering [10].

A follow-up study including more EDs in Germany with adapted inclusion criteria and newly defined and extended goals is already in process of planning. Also, the aspect of increasing awareness within the ED staff will be systematically evaluated. The concept of such an extended screening study could be used as a blueprint for other rare diseases hereafter.

### Limitations

The present study aimed to screen patients for symptomatic AP at three different sites, with a predetermined sample size. However, mostly owing to the COVID-19 pandemic, the study did not recruit as originally scheduled, resulting in a much smaller sample size than planned. As a result, the study’s ability to draw definitive conclusions about the prevalence of AP in the study population was severely limited.

The study’s design also had further limitations. Specifically, the study was conducted in the EDs of (only) three large sites, all headed by experts who had high expertise in the management of porphyria, but this may not be representative. It remains unclear on whether the findings of this study can be extrapolated to other settings. Notably, it was planned originally to include further sites.

Despite these limitations this study matters: It provides valuable information on feasibility conducting a screening study for a rare disease, such as AP, in the clinical setting of EDs. Furthermore and irrespective of the limitations due to the pandemic the study’s findings show that logistical challenges for such screening studies can be overcome by cooperation of a well organized cross-regional network of experts for rare diseases as well as emergency physicians, who see a high number of patients per year. At last, establishing feasible screening protocols for a number of rare diseases for patients who show up with their symptoms in EDs may result in a higher awareness of the ED staff for these patients in future.

## Conclusions

Screening for APs in the ED is feasible and should be considered in all ED patients with (especially recurrent) abdominal pain of unclear origin. A structured education and brief diagnostic protocols may increase awareness of ED staff for certain rare diseases. In the future, EDs with their high number of patients should be part of a network for rare diseases, especially when the symptoms of the underlying and undetected rare disease lead to a likely presentation in an ED, such as in APs.

## Electronic supplementary material

Below is the link to the electronic supplementary material.

Supplementary Material 1

## Data Availability

All relevant data generated or analyzed during this study are included in this published article. If further datasets are requested, these are available from the corresponding author.

## Abbreviations

ADP: 5-aminolevulinic-acid-dehydratase-deficient porphyria
AIP: Acute intermittent porphyria
ALAS1: 5-aminolevulinic-acid synthase 1
AP: Acute porphyria
ED: Emergency department
EP: Emergency physician
GDPR: EU General Data Protection Regulation
GWT-TUD: Gesellschaft für Wissenschafts- und Technologietransfer at Technical University of Dresden
HCP: Hereditary coproporphyria
PGB: Porphobilinogen
SOC: Standard of care
VAS: Visual analog scale
VP: Variegate porphyria
5-ALA: 5-aminolevulinic acid

## References

[1] Stolzel U, Doss MO, Schuppan D (2019). Clinical guide and update on Porphyrias. Gastroenterology.

[2] Stolzel U, Stauch T, Kubisch I (2021). [Porphyria] Internist (Berl).

[3] Diehl-Wiesenecker E, Somasundaram R. 409 Die Porphyrien. In: Suttorp N, Möckel M, Siegmund B, Dietel M, editors. Harrisons Innere Medizin. 20. Auflage ed: ABW Verlag; 2020.

[4] O’Malley R, Rao G, Stein P, Bandmann O (2018). Porphyria: often discussed but too often missed. Pract Neurol.

[5] Gouya L, Ventura P, Balwani M, Bissell DM, Rees DC, Stolzel U (2020). EXPLORE: a prospective, multinational, natural history study of patients with Acute hepatic Porphyria with recurrent attacks. Hepatology.

[6] Anderson KE, Lobo R, Salazar D, Schloetter M, Spitzer G, White AL (2021). Biochemical diagnosis of Acute hepatic Porphyria: updated Expert Recommendations for Primary Care Physicians. Am J Med Sci.

[7] Stein P, Badminton M, Barth J, Rees D, Stewart MF, British (2013). Best practice guidelines on clinical management of acute attacks of porphyria and their complications. Ann Clin Biochem.

[8] Daniels J, Griffiths M, Fisher E (2020). Assessment and management of recurrent abdominal pain in the emergency department. Emerg Med J.

[9] Field MJ, Boat TF, editors. Rare Diseases and Orphan Products: Accelerating Research and Development. The National Academies Collection: Reports funded by National Institutes of Health. Washington (DC)2010. 21796826

[10] Brenner DM, Brandt LJ, Fenster M, Hamilton MJ, Kamboj AK, Oxentenko AS, et al. Rare, overlooked, or underappreciated causes of recurrent Abdominal Pain: a primer for gastroenterologists. Clin Gastroenterol Hepatol; 2022. 10.1016/j.cgh.2022.09.022 36180010

[11] Mockel M, Searle J, Muller R, Slagman A, Storchmann H, Oestereich P (2013). Chief complaints in medical emergencies: do they relate to underlying disease and outcome? The Charite Emergency Medicine Study (CHARITEM). Eur J Emerg Med.

[12] Robert M, Penner B, MD, FRCPC, MSc MB, Fishman M. Evaluation of the adult with abdominal pain. UpToDate®: https://www.uptodate.com/contents/search. 2021.

[13] Bonkovsky HL, Maddukuri VC, Yazici C, Anderson KE, Bissell DM, Bloomer JR (2014). Acute porphyrias in the USA: features of 108 subjects from porphyrias consortium. Am J Med.

[14] Trentzsch H, Werner J, Jauch KW (2011). [Acute abdominal pain in the emergency department - a clinical algorithm for adult patients]. Zentralbl Chir.

[15] Gans SL, Pols MA, Stoker J, Boermeester MA (2015). Expert steering g. Guideline for the diagnostic pathway in patients with acute abdominal pain. Dig Surg.

