# Supplementary material for: Detect Acute Porphyrias in Emergency Departments (DePorED) – a pilot study
Source: Orphanet J Rare Dis. 2023 Jun 12;18:146. doi: 10.1186/s13023-023-02768-5 (PMC10258746; doi:10.1186/s13023-023-02768-5)
Supplement: Supplementary file 1 — Supplementary Material 1 [file 13023_2023_2768_MOESM1_ESM.pptx]

## Slide 1
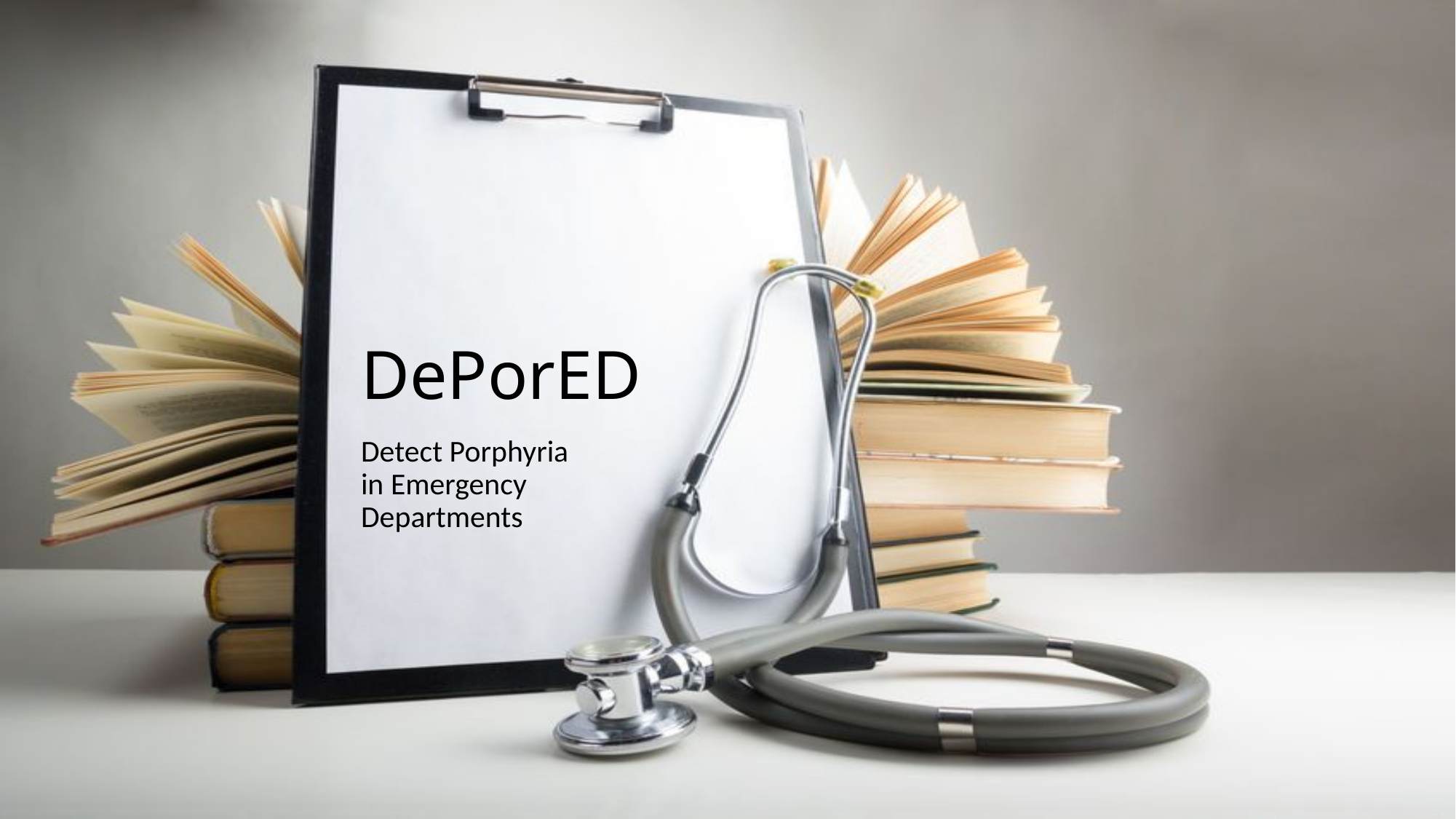

# DePorED
Detect Porphyria in Emergency Departments

## Slide 2
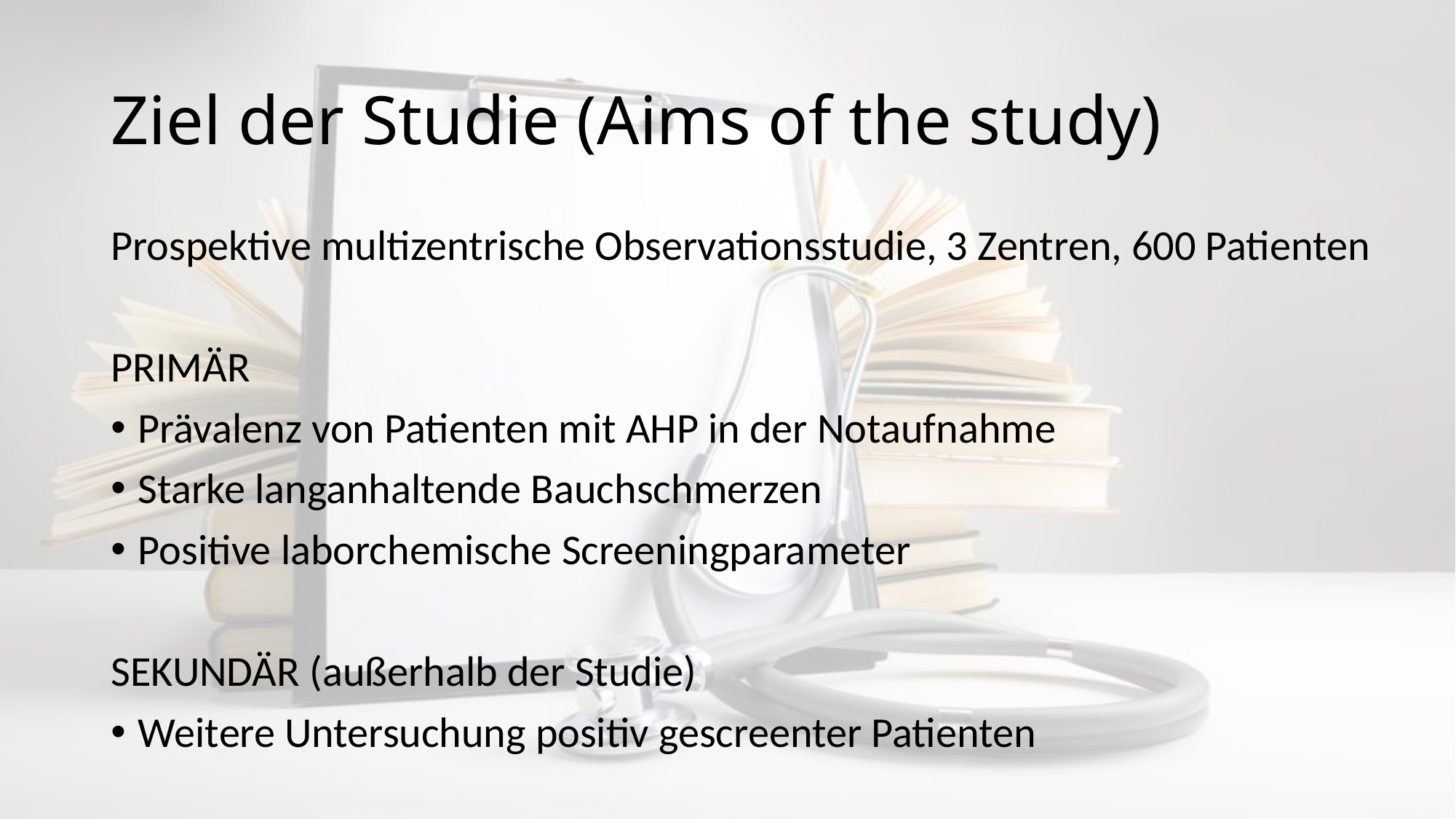

# Ziel der Studie (Aims of the study)
Prospektive multizentrische Observationsstudie, 3 Zentren, 600 Patienten
PRIMÄR
Prävalenz von Patienten mit AHP in der Notaufnahme
Starke langanhaltende Bauchschmerzen
Positive laborchemische Screeningparameter
SEKUNDÄR (außerhalb der Studie)
Weitere Untersuchung positiv gescreenter Patienten

## Slide 3
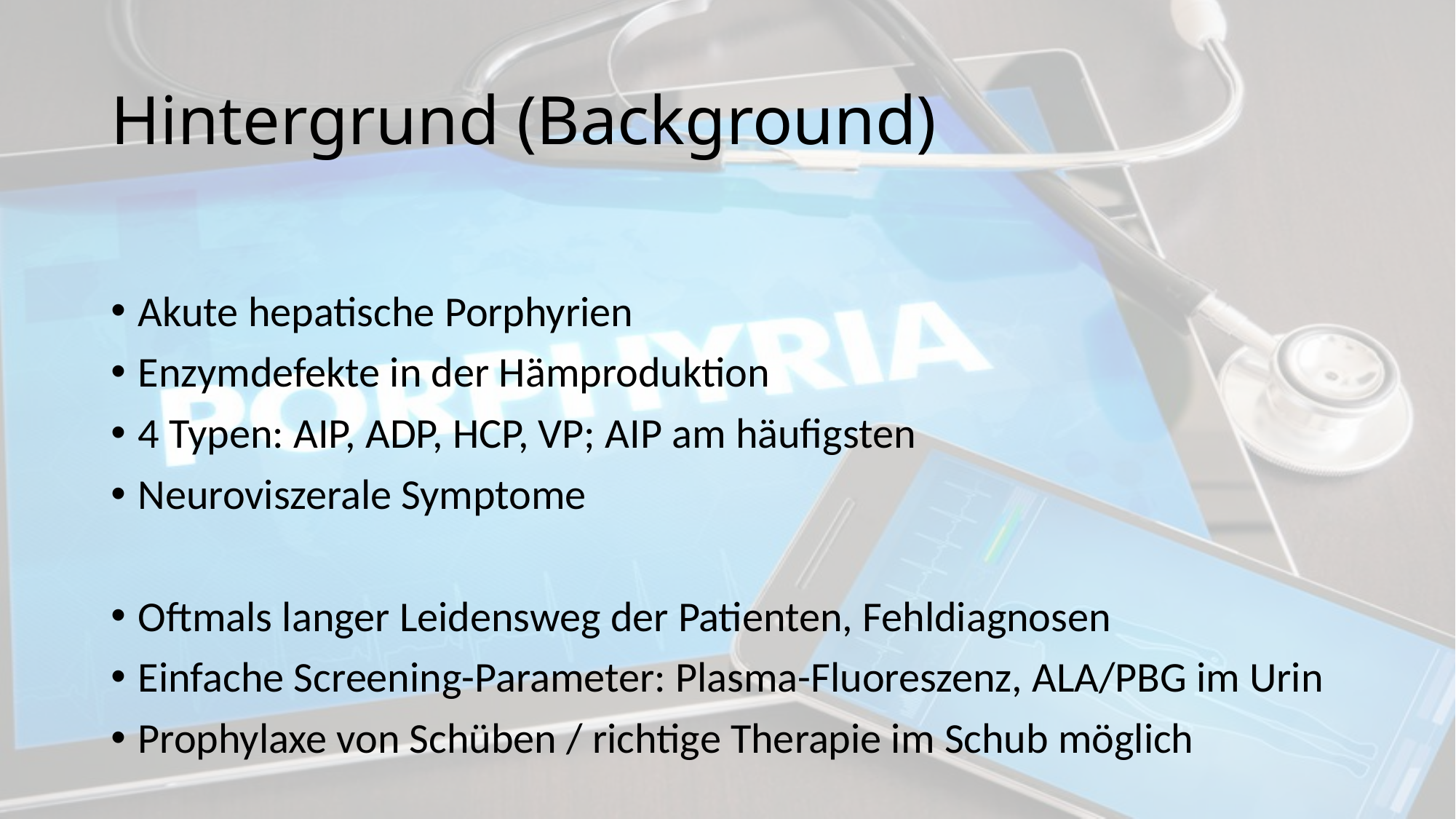

# Hintergrund (Background)
Akute hepatische Porphyrien
Enzymdefekte in der Hämproduktion
4 Typen: AIP, ADP, HCP, VP; AIP am häufigsten
Neuroviszerale Symptome
Oftmals langer Leidensweg der Patienten, Fehldiagnosen
Einfache Screening-Parameter: Plasma-Fluoreszenz, ALA/PBG im Urin
Prophylaxe von Schüben / richtige Therapie im Schub möglich

## Slide 4
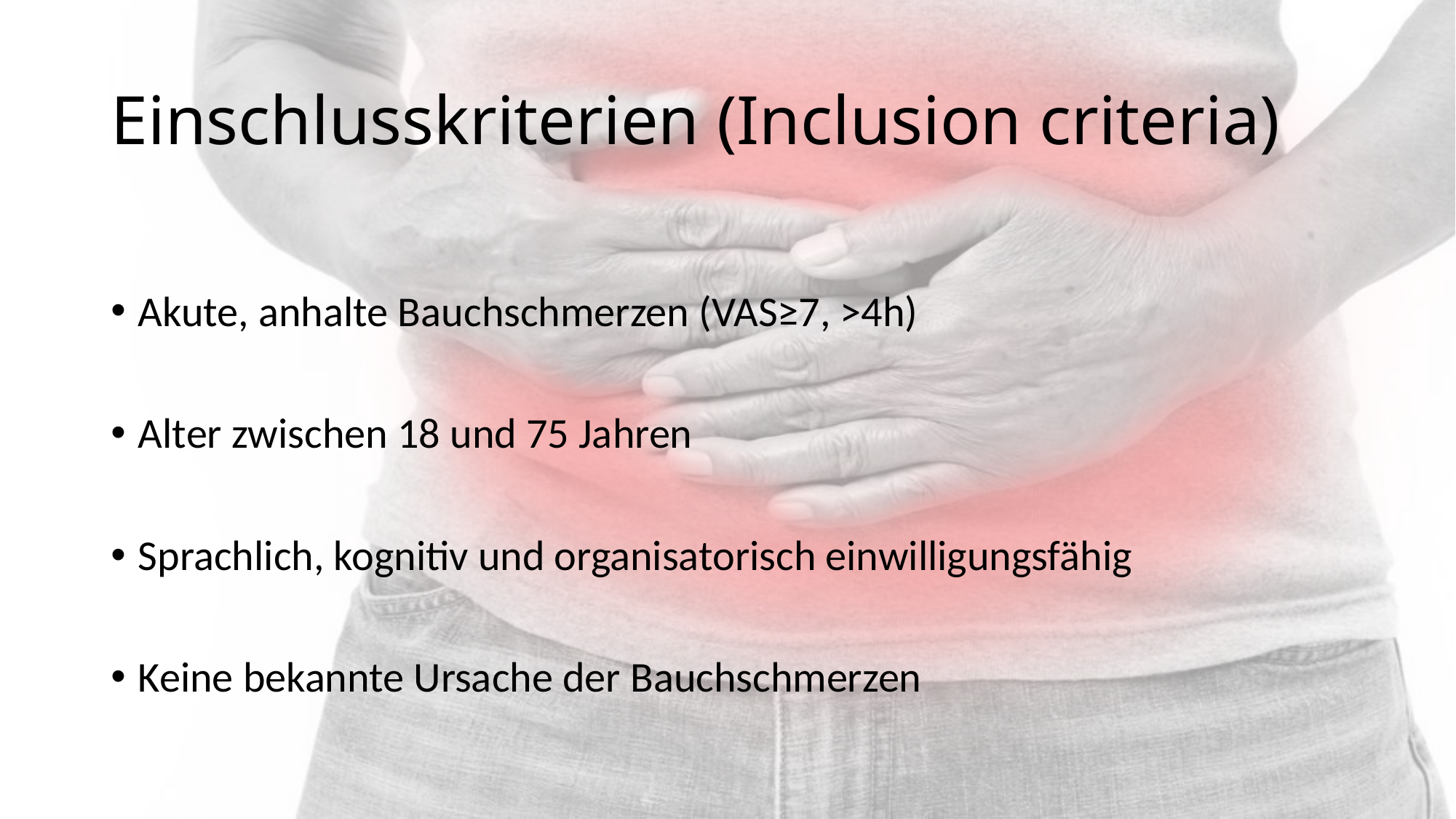

# Einschlusskriterien (Inclusion criteria)
Akute, anhalte Bauchschmerzen (VAS≥7, >4h)
Alter zwischen 18 und 75 Jahren
Sprachlich, kognitiv und organisatorisch einwilligungsfähig
Keine bekannte Ursache der Bauchschmerzen

## Slide 5
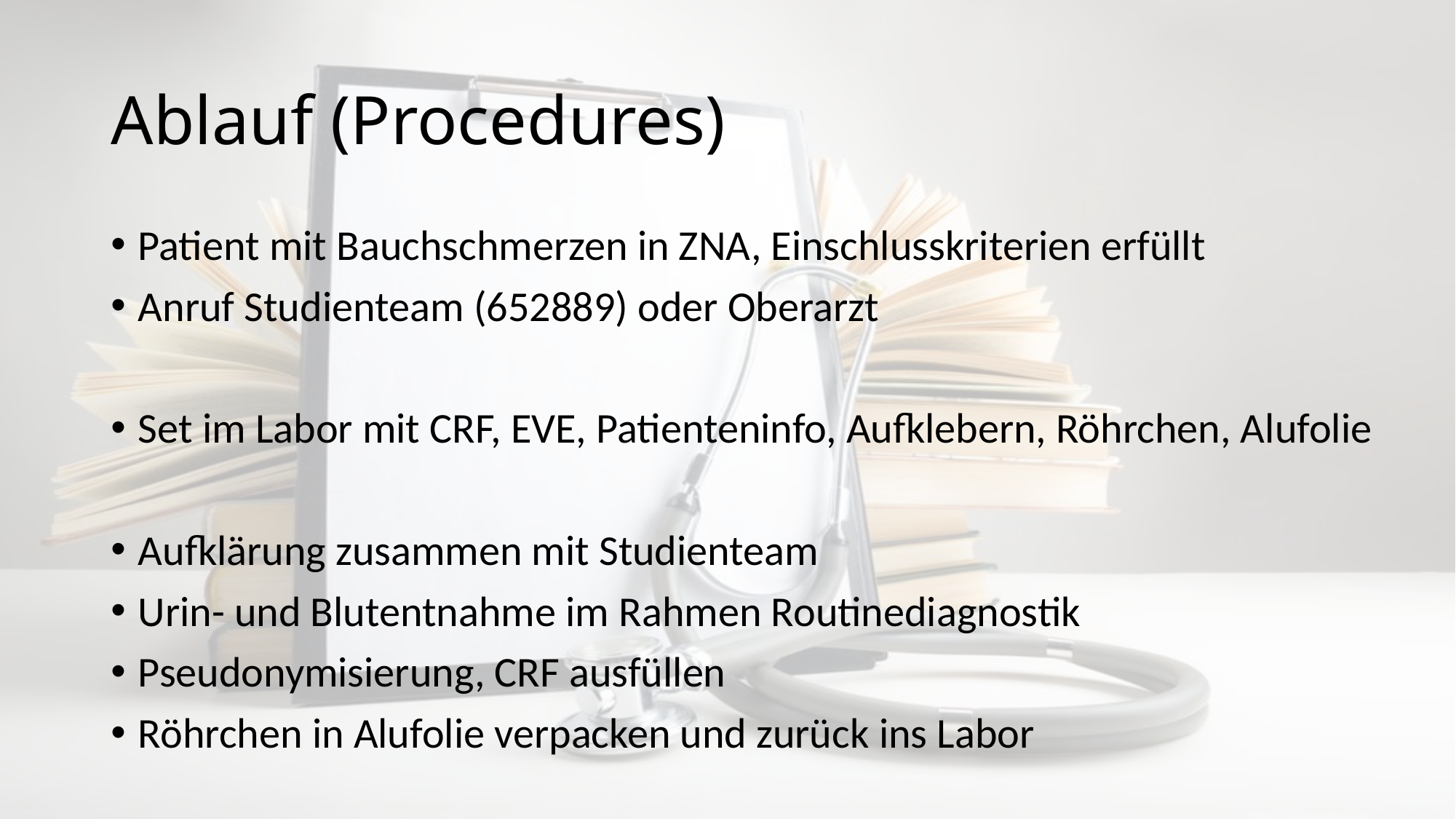

# Ablauf (Procedures)
Patient mit Bauchschmerzen in ZNA, Einschlusskriterien erfüllt
Anruf Studienteam (652889) oder Oberarzt
Set im Labor mit CRF, EVE, Patienteninfo, Aufklebern, Röhrchen, Alufolie
Aufklärung zusammen mit Studienteam
Urin- und Blutentnahme im Rahmen Routinediagnostik
Pseudonymisierung, CRF ausfüllen
Röhrchen in Alufolie verpacken und zurück ins Labor

## Slide 6
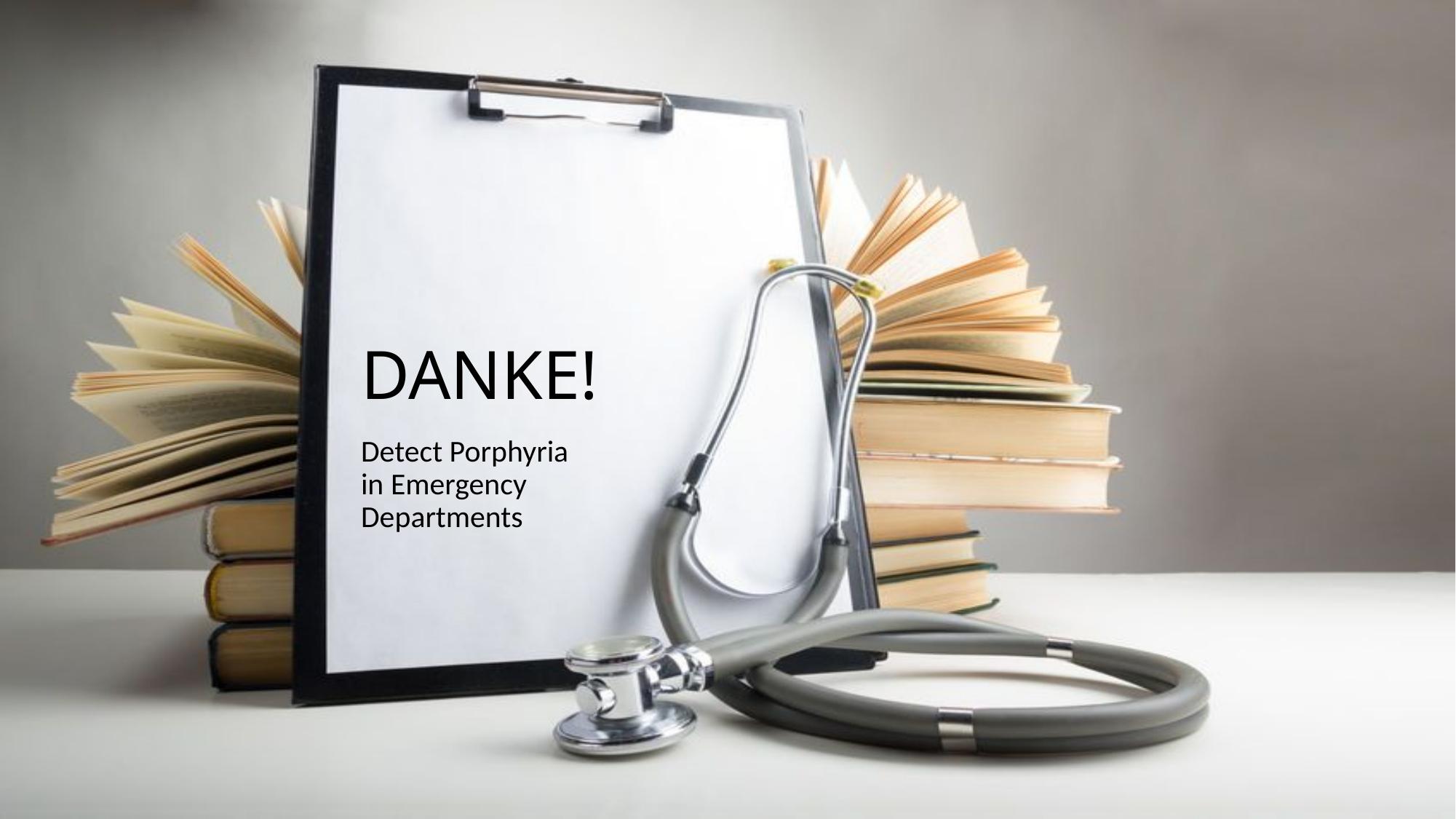

# DANKE!
Detect Porphyria in Emergency Departments
